# Supplementary material for: A role for the C. elegans Argonaute protein CSR-1 in small nuclear RNA 3’ processing
Source: PLoS Genet. 2024 May 14;20(5):e1011284. doi: 10.1371/journal.pgen.1011284 (PMC11125478; doi:10.1371/journal.pgen.1011284)
Supplement: Attachment — Submitted filename: Reviewer responses_PGENETICS-D-23-01143.docx [file pgen.1011284.s010.docx]

**Reviewer #1**: In this manuscript the authors investigate factors that lead to misprocessing (specifically mistermination) of snRNA in C. elegans. They design a clever genetic screen for this, which produced both expected hits and many interesting hits not known to be involved in termination. Particularly interesting, they discover that the Argonaute protein csr-1 is important for correct termination of snRNA. This would be exciting and of major importance if csr-1 actually acted directly in snRNA maturation. However, the authors’ model is that at least part of the effect seems to be due to reduced expression of subunits of the integrator complex due to the fact that csr-1 normally supports expression of some target genes (though their data suggests this is not the only pathway: see below). This mechanism is less interesting- it’s not surprising that a gene that is required for expression of protein coding genes would have secondary effects due to loss of expression. Nevertheless, as a “factoid” the connection of csr-1 to this process, even if indirect, is interesting because it might provide some further insights into how csr-1 mutant phenotypes manifest in development. However, as detailed below, the authors make insufficient attempt to put their observations in the context of what is known already about csr-1. A considerable amount of analysis work would be required to address this, and probably additional high throughput sequencing. Without this I’m not sure it would be suitable for PLoS Genetics.

We thank the reviewer for providing their time and expertise to review our manuscript and help us identify areas of weakness that need to be revised in our manuscript. Please find below our response and actions taken in addressing the reviewer’s point raised which is accompanied by a line number corresponding to the revised clean version of the manuscript.

Major points
**1.** The authors’ model is that loss of the csr-1b protein results in loss of expression of integrator subunits. A lot is known and has been published about the gene expression changes in csr-1 mutants and knockdowns but the authors use their own RNAseq analysis and make no attempt to integrate this with other studies. It is imperative that the authors compare their results with other datasets from the Mello, Cecere and Claycomb labs to see how the expression of the integrator complex is affected (or not) in other analyses. If previous experiments have not revealed gene expression changes in this set of genes above what would be expected by chance, then this will significantly challenge the authors’ model because their results may be false positives.

We apologize for this oversight, and we have now provided comparative context to our RNA-seq data to those from other labs that have done extensive work on CSR-1 mechanisms.

First, we repeated the linear regression analysis in Figure 3b that compared the expression of snRNA downstream genes (an indicator of read-through) using the *csr-1* KO RNA-seq data from the Cerere group (Singh et al., 2021) and found that it also significantly correlates with expression caused by *ints-4* RNAi. This is inserted as **Figure S2d** and discussed in **lines** **193-196**.

Next, for our analysis of transcriptome similarities between Integrator subunit knockdown compared to *csr-1* RNAi knockdown we presented in Figure 3g, we also repeated the analysis using the CSR-1 KO data set from Singh et al. (RNA-seq; 2021) and Claycomb et al., (Microarray; 2009). When comparing genes that were >2-fold up or down-regulated by CSR-1 KO, we also found a significant correlation in expression pattern between CSR-1 KO and across all of the Integrator subunit knockdowns in both datasets. This indicates that the transcriptome similarities we observed between *csr-1* RNAi and Integrator knockdown are also observed in the CSR-1 KO RNA-seq dataset from the Cerere and Claycomb group. This analysis for the CSR-1 KO comparison is incorporated into **Figure S2e** and discussed in **lines** **220-223**.

In interpreting the reviewer’s comment on “how the expression of the integrator complex is affected (or not) in other analyses”, we also examine the mRNA expression level of integrator subunit genes from our study using *csr-1* (RNAi) to those published by the Cerere lab (*csr-1 tm892* mutant with RNA-seq, N=2; PMID: 34108460) and Claycomb lab (*csr-1 tm892* mutant with microarray, triplicate data presented as 1 mean; PMID: 19804758). Across the 3 transcriptome dataset, our RNA-seq data using RNAi showed very similar results in terms of integrator gene fold change with those observed by the Cerere group using the null mutants as determined via RNA-seq. The fold change observed for the microarray data from the Claycomb group was lower for most genes compared to the results from this study and the Cerere study. This may be due to a difference in the sensitivity of the microarray approach vs RNA-seq. This figure is found in **Figure S4c** with accompanying text in **lines 292-294**.

We also want to clarify that our conclusions were primarily based on the analysis of the Ribo-seq data from the Cerere group where it was observed that many of the integrator subunit genes showed a decrease in translational efficiency. As far as we were able to find this publication represents the only instance of Ribo-seq experimental data published to study the effects to translation efficiency upon loss of *csr-1.*

**2.** Related to this, the authors do not make any attempt to correct their data for potential differences in the developmental age of their samples. This is really important because csr-1 RNAi is likely to affect development, causing many genes to vary without them being direct targets of csr-1. Straightforward tools to assess developmental age exist- see for example <https://github.com/LBMC/RAPToR>. These should be applied and any age differences regressed out to see if the results still stand.

We thank the reviewer for this suggestion. We extract our RNA between EV and *csr-1* (RNAi) samples after 72 hours post-synchronized growth starting from L1. In our hands, *csr-1* RNAi does not affect worm development in terms of body length compared to the control and worms develop normally into adulthood. But we recognize that it has been reported that there are underlying differences between the accumulations of oogenesis in the *csr-1* KD worms compared to the WT. We have discussed this potential difference in oogenesis as a limitation factor of our RNA-seq data (**lines 184-186**).

Following the suggestion by the reviewer, we used RAPToR to ‘age estimate’ our RNA sequencing samples to identify potential developmental age differences between EV and *csr-1* treatments*.* This analysis revealed that both EV and *csr-1* RNA samples are highly close in age estimate, EV: 71.84 ±0.08 hours and *csr-1* (RNAi) 71.73 ±0.08 hours. This analysis was performed using ~25,000 transcripts detected in our RNA-seq experiment as the input, and the results suggest that EV and *csr-1* (RNAi) fed worms are largely identical with respect to their age as predicted via transcriptome profile. Shown below are the graphical data as well as the raw data output obtained from R. The graphical data is found in **Figure S2c** of the revised manuscript. We discuss this result in **lines 179-184** of the manuscript.

**3.** A potential issue with the authors’ model that they should consider is how it relates to piRNAs. Csr-1 is supposed to work by preventing aberrant targeting of piRNAs to protein-coding genes. However, piRNA biogenesis, as the authors themselves cite, is promoted by integrator activity. Thus the authors model would predict that primary piRNAs would be depleted in csr-1 mutants. It’s possible that this could occur whilst still having increased piRNA TARGETING as the loss of integrator does not completely block piRNA production and remaining piRNAs could still mis-target. Or alternatively loss of csr-1 leads to increased piRNAs initially but then decreased piRNAs after loss of integrator expression. Regardless, it is clear that the authors need to examine what happens to piRNAs in csr-1 small RNA sequencing. The authors need to
i) Perform small non-coding RNA sequencing under their conditions and examine piRNAs (global levels) and 22G-RNAs targeted to integrator subunits to test if, indeed, csr-1 depletion leads to 22G-RNAs against integrator subunits being induced, and what consequence knockdown of csr-1 has to piRNAs as well as piRNA precursors (do they get misterminated, as expected from the authors’ model?)

We thank the reviewer for their suggestion to provide a link between piRNA and our data on CSR-1. We currently do not have additional resources to fund new RNA-seq experiments as we are in the final year of our funding support before renewal applications.

However, we have analyzed the small RNA sequencing data from the Cerere lab published in their 2021 paper (Singh et al., 2021; Nature Communication 12:3492) which also performed small non-coding RNA sequencing for comparison between N2 and the *csr-1(tm892)* KO strain. As mentioned in a previous response point, the conditions used by Singh et al in the *csr-1* KO experiments show significant similarity in transcriptome profile to Integrator knockdown as determined via mRNA-seq in our analysis (**Figure S2e**). Singh et al. determined that CSR-1 KO results in the global loss of 22G-RNA for the majority of CSR-1 targets. We analyzed this dataset and found that 22G-RNAs targeting Integrator subunits are also depleted. This data indicates CSR-1 is required for the biogenesis of 22G-RNAs targeting the Integrator, and would support the model of CSR-1’s anti-silencing role for Integrator expression via the biogenesis of 22G-RNAs to target germline mRNAs. We have incorporated this data in **Figure S4b** and discussed in **lines 287-290.**

Using the same sRNA dataset, we analyzed the effects of CSR-1 KO on the expression of piRNA. We found that the expression for 2544 piRNAs was significantly altered, surprisingly, almost all of them (2541/2544) were up-regulated in the CSR-1 KO strain. When examining the the global expression of piRNAs regardless of statistical significance, the same trend persists in the CSR-1 KO mutant where the majority of the piRNA are also up-regulated. It has been shown previously by the Mello lab that loss of *csr-1* increases piRNA targeting (Shen et al., 2018; PMID: 29456082); it is also possible that loss of *csr-1* also affects piRNA abundance. With respect to how this relates to Integrator’s role in piRNA maturation, the previous report demonstrating a role for Integrator in piRNA showed that RNAi depletion of *ints-11* results in the decrease in abundance of mature piRNA, and it concludes that the Integrator complex as a whole is required for piRNA cleavage. However, whether the loss of other Integrator subunits also contributes to piRNA defect was not explored. In our study, we curiously see that in the *csr-1(tm892)* mutant, the mRNA, transcription, and translation of *ints-11* were all consistently elevated (**Figure 5b-d**). It is possible that piRNA cleavage activity is an Integrator subunit-specific event and that the lack of reduced expression of piRNA in the *csr-1* mutant could be attributed to the fact that loss of *csr-1* does not decrease *ints-11* expression. As such, it remains to be determined whether the global increase in piRNA expression in the *csr-1(tm892)* mutant is influenced by its effects on *ints-11* expression, or via other potential pleiotropic functions that CSR-1 may exert in the germline that can affect piRNA expression and targeting. We have included this speculation in our discussion in **lines 306-317** and proposed future directions to explore on roles of other Integrator subunits in piRNA processing. The graph illustrating mature piRNA expression change is added as **Figure S4g**

ii) Check in the many small RNA sequencing datasets from integrator mutants to see if piRNAs are affected at all. I don’t think anyone has reported this before but they may not have looked, particularly at piRNA precursor termination defects.

As far as we know, there are no published or preprint studies that performed small-RNA sequencing of Integrator mutants. The study by the Sarkies lab (Beltra et al., 2021 EMBO J) which identified a role of *ints-11* in piRNA processing is the only study that examined small-RNA sequencing via RNAi depletion. The only other next-generation sequencing dataset on Integrator knockdown is mRNA-sequencing performed in the following two studies (Wu et al., 2019 BMC Biology; Gómez-Orte et al., 2019; PLOS GENETICS).

**4.** The authors’ model of an indirect role for csr-1 function in snRNA termination is logical but as they themselves report is not sufficient to explain what csr-1 is doing because csr-1 and integrator are not fully epistatic*. The authors then need to explain what else csr-1 might be doing in this pathway. Is it acting directly on the termination as well? This would be very exciting although difficult to imagine how it might get targeted there. Are there any 22G-RNAs that are csr-1 dependent that are antisense to snRNAs?? This would be a way that snRNAs could be targeted by csr-1. Otherwise, what else is happening? Whilst it is an open ended question I really think that this is an important issue for the authors to try and provide some information about.
*Although… I think that the authors’ epistasis experiment might be confounded by the fact that they do not use complete null mutants for integrator or csr-1, which makes epistasis difficult to interpret. A better test of their model would be to try to restore integrator levels to csr-1 mutants by complementing with transgenes, though I guess that if the entire complex is affected transcriptionally this would be hard (If all subunits are limiting). If I’ve got this correct, the authors may wish to tone down their interpretation of the epistasis experiments by introducing this point as a caveat.

We thank the reviewer for sharing their analysis of our model, and we agree that there may be more that is happening beyond the expression control of Integrator subunits in CSR-1’s role in snRNA processing. We did find that via CSR-1b IP data from the Claycomb lab that CSR-1b binds to endo-siRNA that are anti-sense to snRNA transcripts, though these endo-siRNA bind to the 5’ coding region of snRNA rather than at the 3’ cleavage site. This is in contrast to *csr-1* dependent endo-siRNAs that target histone transcripts in the 3’ region near the stemloop cleavage motifs. However, this does not rule out a direct role for CSR-1b in performing direct cleavage of snRNA as the position of 22G-RNA binding relative to the site of CSR-1 slicing site is not fully understood. We have now included a supplementary figure illustrating IP enrichment of 22G-RNA antisense of snRNA transcripts that are enriched by CSR-1b and emphasize the alternative possibility that CSR-1 could also directly target snRNA via 22G-RNA binding (**Figure S4a**). This alternative possibility is elaborated in our results and discussion section as well as included in our proposed model. (**lines 277-282; 421-424**). We have also toned down our interpretation of the co-depletion experiments and introduced the caveat of not using complete loss of function mutants in the epistatic experiment and that there may still be residual activities of CSR-1 and INTS-4 present after the knockdowns (**lines 267-269**).

Minor point

Csr-1b: I’m not sure that the authors have definitively shown that this isoform is required because their experiments formally only demonstrate that csr1-a is not responsible. If they cannot specifically knock out 1b whilst preserving 1a then they need to tone down their language here to make it clear exactly what their data shows.

We thank the reviewer for their insight and have clarified our interpretation that our data only definitively show that CSR-1a is not involved in snRNA processing but cannot conclusively determine that only CSR-1b is responsible for snRNA processing due to the fact that the knockout mutant used affects both isoforms. As such, we have revised our language to indicate that there remains the possibility the phenotype we observed is attributed to when both isoforms are lost, rather than isoform b specifically. These revisions are found in the text (**lines 147-150; 387-389**). We have also changed our manuscript title to ‘A role for the *C. elegans* Argonaute protein CSR-1 in small nuclear RNA 3’ processing’ to reflect this. In addition, we have removed the following sentence that was in the introduction “Through isoform analysis, we distinguish that the influence of *csr-1* on snRNA processing is specific to the *csr-1b* isoform and requires its catalytic slicing activity”

**Reviewer #2**: Review of manuscript titled “A role for the C. elegans Argonaute protein 1 CSR-1b isoform in small nuclear RNA 3’ processing” by Waddell and Wu.

In the manuscript, the authors use a genome-wide RNAi screen to investigate the regulators of the Integrator complex involved in the maturation of snRNA transcripts. They developed an elegant reporter by fusing GFP downstream snRNA transcript, which will express only in case of misprocessing of snRNA transcript. Among multiple regulators identified by screen, authors focused on Argonaute CSR-1 smaller isoform and showed RNAi of csr-1 or loss of catalytic activity resulted in snRNA misprocessing. They further show that the misprocessing of snRNA due to csr-1 knockdown is additive to the loss of integrator function. They further analysed a previously published dataset and show loss of csr-1 results in decreased translation efficiency of components of integrator complex.
Overall, the study is well done and will be important for the field.

We thank the reviewer for their positive comments on our manuscript as well as suggestions for improvements. Please find below our actions taken in response to each of the concerns raised.

I have a few minor comments.
**1**. Lines 82-85: In the introduction, the authors talk about the role of csr-1 in protection. They should also discuss that, in addition to its protective function, csr-1 has now been shown also to cleave mRNA.

We thank the reviewer for this reminder. We have now described in the introduction a role for CSR-1 in the embryo cleavage of maternal mRNA for clearance and removal and cited the corresponding study from Quarato et al. (PMID:33664268). This is found in **lines 85-86** of the introduction.

**2.** In figure 3, the authors show the presence of an increased number of alternatively spliced transcripts. Can they comment on how their levels are compared to wt?

This is a great suggestion by the reviewer and we have now examined the expression of genes that were alternatively spliced after *csr-1* RNAi. Of the 391 total AS events, 60% of the genes were differentially expressed (232). Of these 232 genes, 139 were up-regulated and 93 were down-regulated. This 60% differential expression rate is higher than the global effect *csr-1* RNAi has on the transcriptome where 31% of the transcriptome showed were differentially expressed (FDR<0.05, no fold change cut off). This suggests that *csr-1* RNAi induced AS transcript may also cause alterations to its mRNA gene expression. We have commented on this relationship between AS and gene expression in **lines 207-213**.

**3.** Figure 5 legend, 5a- authors have written Chip-seq data, I presume it is a typo mistake as they are showing 22G-RNAs, so it should be small RNA-seq. Please check.

We apologize for this mistake, we have now corrected the typo to read “small RNA-sequencing” in the figure legend. This correction is found in **line 794**.

**4.** Though authors have mentioned N in their methods, it will be useful to mention n=? for reader experience in all the figures with bar graph, number of transcripts in scatter plots and heat maps in fig 3, either as part of the figure or in the legend. Also, showing individual data points in the bar graphs wherever feasible is better.

We thank the reviewer for their input and have updated N values to the figure legend of scatter plots and heatmaps where the graph does not explicitly indicate the number of genes. These changes are found throughout the figure captions. We have also revised all of our bar graphs in the manuscript to now indicate the individual data points such that the readers can visualize the sample numbers and variances; these changes can be seen in all the bar graphs of the revised manuscript figures.

**5.** To show the impact of loss csr-1 on integrator complex, authors have analysed previously published data for RNA-seq, GRO-seq and Ribo-seq for csr-1 KO, where they see reduced translation of integrator complex. Considering, they also see that rescue by catalytic inactive csr-1 also doesn’t rescue misprocessing, it will be interesting to see if the same impact of integrator transcripts is also seen CSR-1 catalytic mutant. They can analyse the data for csr-1SIN from the same publication as that for KO.

We thank the reviewer for this suggestion to strengthen our data regarding the requirement of CSR-1 catalytic activity on the Integrator transcript. We have now analyzed the RNA-seq, Gro-Seq, and Ribo-Seq data from the CSR-1 catalytic dead mutant (termed ‘CSR-1 ADH’ by the Cerere lab) from the Singh et al. paper and show its effect on Integrator genes in parallel with the CSR-1 KO strain (**Revised Figure 5b-d**). In general, the effects of CSR-1 KO and CSR-1 ADH are very similar for all 3 types of sequencing approaches. To quantify this similarity, we performed linear regression analysis for the 3 sequencing approaches for the 12 Integrator genes and found a correlation value of 0.82 for mRNA-seq, 0.85 for Gro-seq, and 0.81 for Ribo-seq (**Figure S4d-f**). This data reinforces our *csr-1*^SIN^ data which illustrates the requirement of CSR-1 catalytic activity for both snRNA processing and Integrator transcript regulation. We have now incorporated this dataset in the results sections (**lines 298-303**).

**6.** Finally, in the genome wide screen, authors identify in addition to csr-1 many other possible regulators which broadly categorising seems to be not only csr-1 but quite a few other members of RNAi pathway, nuclear pore proteins, ribosomal proteins among others. In the discussion, authors do talk about nuclear pore proteins, but it will be worth discussing other regulators, especially those that belong to RNAi pathways like mut-16, dcr-1, alg-4, rde-4 identified in the screen. It is possible that regulation by small RNAs might be more complex and extend beyond just csr-1.

We thank the reviewer for noticing other RNAi regulators identified in our screen. We did not previously discuss these genes as we did not verify their levels of endogenous misprocessing via qPCR. But we now recognize that there may be a broader significance and interest of these genes in that additional RNAi mechanisms such as those involving 26G-RNA may also play a role in snRNA processing in addition to the CSR-1 mediated 22G-RNA pathway. We have provided a brief discussion on this in **lines 457-464**, emphasizing that the full mechanism may be more complex and may involve endogenous RNAi pathways that go beyond CSR-1.

**Reviewer #3**: In this manuscript, Waddell and Wu investigate the regulation of snRNA 3’ processing by the essential C. elegans Argonaute CSR-1. The authors use a clever GFP-based reporter for snRNA processing to observe defects in snRNA 3’-end termination. Using this system, they conduct a genome-wide RNAi screen and identify csr-1 among the 47 candidate genes. They show clear snRNA misprocessing in csr-1 mutant conditions, which is specific to the csr-1b isoform and requires CSR-1 catalytic slicer activity. The authors further hypothesize that components of the Integrator complex are disrupted in csr-1 mutants. To explore this hypothesis, they compare transcriptomic changes between csr-1 RNAi and RNAi targeting ints-4, an Integrator subunit. The comparison shows dysregulation at the same snRNA loci and similar overall transcriptomic profiles. To examine the genetic interaction between csr-1 and ints-4, the authors degrade INTS-4 and simultaneously knockdown csr-1 using RNAi. snRNA misprocessing defects observed upon INTS-4 degradation are exacerbated upon csr-1 RNAi, potentially due to CSR-1 regulation of multiple Integrator subunits.
To analyze the impact of CSR-1 on Integrator expression, they examine published RNA-seq, GRO-seq and Ribo-seq data. With unchanged transcription and mRNA abundance, Integrator subunits exhibit reduced ribosome occupancy by Ribo-seq. This reduced translation leads to decreased germline INTS-4 abundance, as measured by fluorescence microscopy. In summary, this study identifies a role for the CSR-1 22G RNA pathway in regulation of snRNA processing. Most of the findings are well supported by data. However, the mechanism by which this regulation occurs requires more examination. This study would be appropriate for publication in PLoS Genetics upon completion of some essential revisions. The proposed model of interaction between CSR-1 and Integrator requires more robust supporting data and a more cohesive description.

We thank the reviewer for their thorough evaluation of our manuscript and for providing key areas of weakness in our study that needed improvements. Please find below our response and actions taken for each of the concerns raised.

Major criticisms:

**1.** The authors propose a model by which CSR-1b binds 22G RNAs complementary to Integrator subunits. This complex, requiring CSR-1 slicer activity, then binds to mRNAs encoding Integrator subunits and activates translation. To support the proposed model, I suggest the authors consider a set of additional experiments.

a) To strengthen the claim that csr-1 mutants have reduced Integrator protein abundance, I suggest a more robust method such as quantitative western blots. When fluorescence microscopy is used to quantify tissue specificity of protein expression, germline tissue may be extruded and imaged to minimize background autofluorescence shown as in figure 5e.

We thank the reviewer for this suggestion. We did not perform Western blot to assess INTS-4 abundance as the observed change in the *csr-1(tm892)* mutant was only found in the germline, and this difference in expression may not be detected using whole-body lysate. We instead followed the second suggestion of the reviewer and performed germline dissection to measure INTS-4::mKate2 expression between wildtype and the *csr-1(tm892)* mutant. In the dissected germline, we observed the same decrease in INTS-4::mKate2 fluorescence in the *csr-1(tm892)* mutant compared to the wildtype, which strengthens our initial *in vivo* observations in the intact whole animal. This new data is incorporated as **Figure S5b** and discussed in **lines 329-334**. We do recognize the limitation of relying on fluorescence to determine protein expression and this method is not as robust compared to quantitative Western Blot, as such, we have discussed this caveat as a potential weakness in **lines 334-336**.

b) To strengthen the claim that CSR-1 regulates multiple Integrator components, it would be helpful to include protein level analysis of additional Integrator subunits.

We thank the reviewer for this suggestion. We decided to interrogate the effects of *csr-1* null on INTS-6 expression given that Ribo-Seq data suggests a decrease in translation efficiency of *ints-6* in the *csr-1(tm892)* mutant (**Figure 5d**). We crossed a strain expressing *ints-6p*::INTS-6::eGFP (JCP341) into the *csr-1(tm892)* background (WM182) to determine the effects of *csr-1* loss of function on INTS-6 expression in the germline compared to the wildtype. We observed that similar to INTS-4::mKate2, the loss of *csr-1* led to a significant decrease in INTS-6::eGFP fluoresce compared to the wildtype in the germline. This data corroborates the Ribo-seq data and indicates that loss of *csr-1* decreases the germline expression of INTS-4 and INTS-6. We have included the new experiment in **Figure 6a** and discussed these results within the manuscript in **lines 337-341**.

c) Throughout the paper, the proposed mechanism should be more clearly written. A model figure would be helpful.

We apologize for this omission and thank the reviewer for this suggestion. We have now added a new **Figure 6c** to illustrate our proposed model. The description of the model is found in text in lines **369-372**.

**2.** When comparing the transcriptomic changes observed in csr-1 RNAi and ints-4 RNAi, the authors compare their csr-1 RNAi dataset to the ints-4 RNAi dataset published from another lab. To minimize batch effects, I suggest the sequencing of ints-4 RNAi alongside csr-1 RNAi from the same replicates. Alternatively, the authors could compare the samples collected from both studies and show that batch effects are minimal. A PCA of all samples shown in figure 3G alongside both empty vector controls would be sufficient.

We thank the reviewer for this suggestion and we now recognize the need to discuss limitations in our manuscript when comparing transcriptomic data from different labs. We performed PCA analysis of EV samples between our study and the RNA-seq data published by Gómez-Orte et al. using the normalized counts as the input. As seen in the figure below, there is a batch variation between the EV samples (accompanied **figure** **panel a** below), this is likely contributed by factors including potential variants of the N2 strain between labs, but is likely heavily influenced by the timing of the RNA extraction. In our experiment, we extracted the RNA 72h post L1 grown at 20°C, while the Gómez-Orte paper extracted RNA 144h post L1 grown at 15°C. These factors are likely a major contributor to different baseline levels of transcript abundance between the 2 EV samples. However, we still believe our observation that *csr-1* and *ints* RNAi exhibit similar transcriptome effects to be valid since we are normalizing each dataset with their corresponding EV controls, which would largely account for baseline variations in conditions for each experiment. To provide some confidence in this comparison, we compared the *csr-1*/EV transcriptome data from this study to another previous study that measured the effects of *ints-4* RNAi on the transcriptome (RNA was extracted from L3/L4 stage of this study; PMID: 31311534). Again, we observed batch variations in the EV samples (accompanied **figure panel b** below), but the pattern and correlation of gene expression change between *csr-1* and *ints-4* were consistent between both studies (accompanied **figure panel c** below). This suggests that while batch variations exist regarding the baseline EV values between the experiments, the relative fold change in gene expression between *csr-1* and *ints-4* are consistent between 2 independent studies. We have now explained this caveat in our data comparison in the manuscript (**lines 225-232**) and provided the correlation analysis between *csr-1* and *ints-4* data from the 2 different studies shown now in **Figure S2f**.

Furthermore, in responding to another comment raised by reviewer 1 above, we also compared the transcriptome of the *csr-1* null mutant published by the Cerere and Claycomb groups to the transcriptome of Integrator subunit knockdown and found significant correlations across all Integrator subunits from both studies (**Figure S2e**). Collectively, these comparisons show that while batch effect exists for the controls used between different studies, the net effect of *csr-1* perturbation when normalized to their respective control produces a transcriptome profile that is similar to Integrator subunit knockdown.

**3.** Data shown in figure 4 suggests that csr-1 RNAi and INTS-4 degradation have additive impacts on snRNA misprocessing. The authors suggest that this may be due to CSR-1 regulating multiple Integrator subunits. For this statement to be possible, it is important to show that reduction of other Integrator subunits by RNAi also exacerbates the INTS-4 degradation phenotype. I suggest the completion of an additional condition in Figure 4e which uses INTS-4::degron +/- auxin or +/- additional Integrator subunit RNAi. If misprocessed U2 increases upon loss of an additional Integrator subunit, it is plausible that CSR-1 regulation of other Integrator subunits makes the phenotype additive with INTS-4 degradation.

We thank the reviewer for this valuable suggestion. We have now performed a new experiment where we simultaneously knocked down INTS-4 via auxin and *ints-6* via RNAi to assess the effects on snRNA processing. We chose *ints-6* as the Ribo-Seq data also showed a decreased expression of this subunit in the *csr-1(tm892)* strain which is supported by a decrease in INTS-6::eGFP levels.

We found that co-depletion of subunits 4 and 6 exacerbated the effects of snRNA misprocessing compared to the depletion of subunit 4 or 6 alone. Interestingly, the effects were of a synergistic nature and we suspect that this may be due to the fact that the loss of a single subunit may also impact the activity of its interacting subunit; INTS-4 has been shown to anchor INTS-9 and 11 as part of the catalytic subunit and INTS-6 associates with subunits 2, 5, and 8 to form the backbone core. These results support our model where CSR-1 contributes to snRNA processing by regulating multiple Integrator subunits. We have included this data as a new **Figure 6b** and the accompanying revision to text is found in **lines 345-353, 435-436**.

Minor comments:

Several small typos
**1.** I do not see a reference for figure 2d or 5d in the text.

We apologize for this oversight, we have now added reference to Figure 2d (**line 147**) and 5d (**line 297**) in the text.

**2.** Line 195 “transcriptome profile of between csr-1 and Integrator” should have “of” or “between” removed.

This typo has been corrected, we thank the reviewer for noticing this error (**line 214**).
